# Fomentations, Lotions, and Bed-Sores

**Published:** 1887-02-19

**Authors:** 


### TILL THE DOCTOR COMES.

[Inquiries and Communications for this column should be addressed, "Physician," care of the Editor of THE HOSPITAL.]

### XI.—FOMENTATIONS, LOTIONS, AND BED-SORES.

*Hot Fomentations.*—Must be hot and well wrung out. New flannel is the best material. Put a short round towel over an empty basin; place the dry flannel in the towel, and pour over it plenty of boiling water. Quickly wrap the towel round the flannel, and twist the two ends of the towel (into each of which a wooden roller may be first inserted) in opposite directions, so as to squeeze out all the water. Apply it immediately, and cover it and the adjoining parts with a piece of mackintosh, or with another thick piece of dry flannel, doubled so as to thoroughly overlap the wet flannel on all sides. These fomentations must be changed about every four hours, or as soon as they feel cold.

*Poppy Fomentations.*—Crush two poppy heads and boil well in two pints of water; strain, wring out a flannel in the hot liquid, and apply in the same way.

*Turpentine Fomentations.*—Sprinkle a tablespoonful of turpentine over the surface of the hot flannel prepared as for hot fomentations. Keep it on about twenty minutes, or till the skin is quite red.

*Laudanum Fomentations.*—Sprinkle in the same way a teaspoonful of laudanum over the surface of the hot flannel. These may be applied continuously like the hot fomentations as long as there is pain, being renewed when cold.

*Cold Applications.—Ice-Bag.*—Half fill a thin bladder or india-rubber bag with small pieces of ice: if filled, it does not adapt itself to the part. It must be renewed before the ice is quite melted. Used to allay inflammation in a wounded part.

*Evaporating Lotions.*—Made with vinegar or spirit, in the proportion of a wineglassful to half a pint. They must not be covered, but left to evaporate quickly, as they thus act by keeping down the temperature of an inflamed part. The bed during their use must be protected by waterproof sheeting. One or two folds of rag must be laid over the part, and kept constantly wet with the lotion. It is a common mistake to use too many folds of rag; when this is done, the lower layers get quite hot, as their lotion cannot evaporate: they thus keep the part hot instead of cold. Any spirit—as gin or eau de cologne—will answer the purpose. Such lotions are very serviceable in reducing inflammation, but not so efficient as the ice-bag, which should be reserved for extreme cases, as a wounded joint, or an injury to the head, when inflammation of the brain is dreaded.

*Water Dressing.*—Dip a piece of lint of the requisite size in hot water, apply to the part, and cover with a piece of gutta-percha tissue *larger* than the lint, so that it will completely overlap it on all sides ; bandage it on firmly. Thus applied, the lint ought to keep warm and moist for twenty-four hours, or longer. Its action is that of a mild poultice, and it generally succeeds that dressing on a discharging wound.

*Lotions.*—Applied in the same way, dipping the lint into the lotion, instead of into hot water. The lint soon gets warm. Carbolic acid, sanitas, boracic acid, etc., are used in this way as disinfectants to keep down smell and discharge ; sulphate of zinc, alum, nitric acid, etc., as stimulants to indolent, slowly healing wounds. It is always better to use a disinfectant than water dressing alone, as any smell is thus much more surely avoided ; boracic acid is the least irritating disinfectant.

*Ointments.*—Must be spread on lint, covered with another piece of dry lint, and bandaged. They are exceedingly varied, and of different uses. Zinc ointment and vaseline are in most common use for ordinary dressings : the latter, however, seems often to irritate the surrounding sound skin. Iodoform ointment cannot be too highly praised for its usefulness in taking away the smell from foul wounds : it is far more serviceable than the disinfecting lotions, though the latter are more generally used. Some people, however, object to the smell of iodoform, which is a very peculiar one, and it also stains linen permanently.

*Liniments.*—Pour a little liniment into the palm of the hand, and rub briskly into the part till the hand is dry. In using strong sedative liniments, as aconite or belladonna, or irritating liniments, as croton oil, a little of the liniment must be poured on a small piece of flannel, and then rubbed into the desired part. Too much of these strong preparations must not be used at once.

*Bed-sores.*—Much may be done to avoid bed-sores : by frequent change of position ; by great cleanliness, smoothness of the sheets, and dryness ; by sponging and powdering with starch powder twice a day ; by keeping the bed free from crumbs, etc. ; by the skilful arrangement of pillows and timely use of air-cushions or water-pillows ; by rubbing the skin over exposed parts with brandy, eau de cologne, or spirit and water, and painting them with collodion, or a lotion composed of equal parts of tincture of catechu and dilute solution of acetate of lead. If the skin becomes red, these remedies must be discontinued, and zinc ointment used, with pads so applied as to relieve the pressure on the painful part. Any further stages must be brought under the notice of the medical man.

*Report for Doctor.*—The nurse should always be able to give the doctor an *exact* report of her patient's condition since his last visit. For this purpose, she should make notes of the following points on paper :—Quantity of food : nature, and times at which it was taken ; times of administering medicines ; temperature (if necessary) at different times ; how long he has slept ; how often the bowels have been moved, etc. She must also note, in many cases, the nature of the breathing—the expression of the countenance, as to the amount of pain, etc. ; whether there was delirium, and of what kind—low and muttering, noisy and excited, etc. Any matter of a peculiar kind from the stomach, bowels, etc., must be saved for the doctor's inspection. These directions will be valuable in almost any serious disease, but for any particular disease, she should ask the doctor whether there are any points on which he may desire to be informed.

